# Towards a better solution to the shortest common supersequence problem: the deposition and reduction algorithm

**DOI:** 10.1186/1471-2105-7-S4-S12

**Published:** 2006-12-12

**Authors:** Kang Ning, Hon Wai Leong

**Affiliations:** 1 Department of Computer Science, National University of Singapore, Science Drive, Singapore 117543, Singapore

## Abstract

**Background:**

The problem of finding a Shortest Common Supersequence (SCS) of a set of sequences is an important problem with applications in many areas. It is a key problem in biological sequences analysis. The SCS problem is well-known to be NP-complete. Many heuristic algorithms have been proposed. Some heuristics work well on a *few *long sequences (as in sequence comparison applications); others work well on many *short *sequences (as in oligo-array synthesis). Unfortunately, most do not work well on *large SCS instances *where there are *many*, *long *sequences.

**Results:**

In this paper, we present a *Deposition and Reduction (DR) algorithm *for solving large SCS instances of biological sequences. There are two processes in our DR algorithm: *deposition *process, and *reduction *process. The deposition process is responsible for generating a small set of common supersequences; and the reduction process shortens these common supersequences by removing some characters while preserving the common supersequence property. Our evaluation on simulated data and real DNA and protein sequences show that our algorithm consistently produces the best results compared to many well-known heuristic algorithms, and especially on large instances.

**Conclusion:**

Our DR algorithm provides a partial answer to the open problem of designing efficient heuristic algorithm for SCS problem on many long sequences. Our algorithm has a bounded approximation ratio. The algorithm is efficient, both in running time and space complexity and our evaluation shows that it is practical even for SCS problems on many long sequences.

## Background

The problem of finding a *Shortest Common Supersequence *(SCS) of a given set of sequences is a very important problem in computer science, especially in computational molecular biology. The SCS of a set of sequences can be stated as follows: Given two sequences *S *= *s*_1_*s*_2_...*s*_*m *_and *T *= *t*_1_*t*_2_...*t*_*n*_, over an alphabet set Σ = {σ_1_, σ_2_,...,σ_*q*_}, we say that *S *is the *subsequence *of *T *(and equivalently, *T *is the *supersequence *of *S*) if for every *s*_*j*_, there is  for some 1 ≤ *i*_1 _<*i*_2 _< ... <*i*_*m *_≤ *n*. Given a finite set of sequences *S *= {*S*_1_, *S*_2_,...,*S*_*k*_}, a *common supersequence of S *is a sequence *T *such that *T *is a supersequence of *every *sequence *S*_*j *_(1 ≤ *j *≤ *k*) in *S*. Then, a *shortest common supersequence *(*SCS*) *of S *is a supersequence of *S *that has *minimum *length. In this paper, we shall assume that *k *is the number of sequences in *S*, *n *is the length of each sequence, and *q *= |Σ| is the size of the alphabet.

The SCS problem has applications in many diverse areas, including data compression [1], scheduling [2], query optimization [3], text comparison and analysis, and biological sequence comparisons and analysis [4,5]. As a result, the SCS problem has been very intensively researched [6,7]. One basic result is that the SCS of *two *sequences of length *n *can be computed using dynamic programming in *O*(*n*^2^) time and *O*(*n*^2^) space (see, for example, [8]). There are also several papers that reported improvements on the running time and space required for dynamic programming algorithms [7]. For a fixed *k*, the dynamic programming algorithm can be extended to solve the SCS problem for *k *sequences of length *n *in *O*(*n*^*k*^) time and space. Clearly, this algorithm is not practical for large *k*. The general SCS problem on arbitrary *k *sequences of length *n *is well-known to be NP-hard. In fact, Jiang and Li [8] showed that even the problem of finding a constant-ratio approximation solution is also NP-hard.

A trivial algorithm, called Alphabet [6] gives an approximation ratio of *q *= |Σ|. In practice, it is well known that heuristic algorithms produce results that are better than the Alphabet algorithm. Many heuristic algorithms have been proposed for the general SCS problem, including Alphabet [6], Majority Merge [8], Tournament [9], Greedy [9], and Reduce-Expand [6]. Several heuristic algorithms were also proposed specifically for computing the SCS of DNA sequences (with alphabet size of 4). These include Min-Height [10], Sum-Height [10] heuristics. (Interestingly, the Majority Merge [8] and Sum-Height [10] heuristic are the same algorithm.) Recently, we [11] proposed *look-ahead extensions *of these heuristics, as well as a post-processing reduction procedure and studied the performances of these algorithms on DNA sequences to be used for the synthesis of oligo-array.

This paper focuses on algorithms for solving *large SCS instances*. By *large SCS instances*, we mean SCS instances *S *in which

(a) the sequences in *S *are *long *(*n *is 100 to 1000),

(b) there are *many *sequences (*k *is 100 or more), and

(c) the alphabet set may be *big *(*q *is 20 for protein sequences).

Large SCS instances arise more frequently in the post-genome era in biological applications dealing with DNA and protein sequences.

In this paper, we propose to solve large SCS instances with our *Deposition and Reduction *algorithm (DR). The DR algorithm is based on the post processing algorithm that we have proposed on the SCS problem for DNA oligos [11]. The DR algorithm is suitable for solving large SCS instances – for example, SCS instances with up to 5,000 DNA and protein sequences each of length 1000. We present experimental evaluation using simulated data and real DNA and protein sequences to show that our DR algorithm outperforms the other heuristic algorithms for the SCS problem on these large SCS instances.

### Previous research of the SCS problem

We now present a brief survey of several heuristic algorithms for the SCS problem. Due to space limitation, we will focus only on algorithms that we have included in our comparative study.

Let *S *be any instance of the SCS problem and let *CS*_*A*_(*S*) be the supersequence of *S *computed by a heuristic algorithm *A*. Let *opt*(*S*) denote an optimal solution for the instance *S*. Then, we say that *A *has an approximation ratio of λ if |*CS*_*A*_(*S*)|/|*opt*(*S*)| ≤ λ for all instances *S*.

#### Alphabet algorithm [6]

The Alphabet algorithm is a very simple algorithm. Let *S *be a set of sequences of maximum length *n *over the alphabet Σ = {σ_1_, σ_2_,...,σ_*q*_}, then the Alphabet algorithm outputs a common supersequence of (σ_1_σ_2_...σ_*q*_)^*n*^. The Alphabet algorithm has an approximation ratio of *q *= |Σ|. The time complexity of the Alphabet algorithm is O(*qn*). There have also been modifications of the Alphabet algorithm that uses information from *S *to "remove" redundant characters in (σ_1_σ_2_...σ_*q*_)^*n*^. These methods improve the performance in practice, but not in the worst case approximation ratio of *q*.

#### Majority-Merge algorithm [8]

The Majority-Merge algorithm (MM) is a simple, greedy heuristic algorithm. Suppose we analyze every sequence from left to right, the *frontier *is defined as the rightmost characters to be analyzed. Initially, the supersequence *CS *is empty. At each step, let *s *be the majority among the "*frontier*" characters of the remaining portions of the sequences in *S*. Set *CS *= *CS*||*s *(where "||" represent concatenation) and delete the "*frontier*" *s *characters from sequences in *S*. Repeat until no sequences are left. This algorithm is the same as the **Sum Height algorithm (SH) **proposed in [10]. This algorithm does *not *have any worst-case approximation ratio, but performs very well in practice. The time complexity of the Majority-Merge algorithm is O(*qkn*).

#### Greedy and Tournament algorithms [12]

The Greedy algorithm (GRDY) and Tournament algorithm (TOUR) studied in [12] are two variations of an iterative scheme based on combining "best" sequence pairs. Given any pair of sequences, *S*_*i *_and *S*_*j*_, an optimal supersequence of the pair, denoted by SCS(*S*_*i*_, *S*_*j*_), can be computed in *O*(*n*^2^) using dynamic programming. The Greedy algorithm first chooses the "best" sequence pair – the pair that gives the shortest SCS(*S*_*i*_, *S*_*j*_). Without loss of generality, we assume that these two sequences are *S*_1 _and *S*_2_. The algorithm then replaces the two sequences *S*_1 _and *S*_2 _by their supersequence, SCS(*S*_1_, *S*_2_). The algorithm proceeds recursively. Thus, we can express it as follows:

Greedy(*S*_1_, *S*_2_,...,*S*_*k*_) = Greedy(SCS(*S*_1_, *S*_2_), *S*_3_,...,*S*_*k*_).

The Tournament algorithm is similar to the Greedy algorithm. It builds a "tournament" based on finding *multiple *best pairs at each round and can be expressed schematically as follows:

Tournament(*S*_1_, *S*_2_,...,*S*_*k*_) = Tournament(SCS(*S*_1_, *S*_2_), SCS(*S*_3_, *S*_4_),...,SCS(*S*_*k*-1_, *S*_*k*_)).

Both Greedy and Tournament algorithms have *O*(*k*^2^*n*^2^) time complexity and *O*(*kn + n*^2^) space complexity. Unfortunately, it was shown in [9] that both Greedy and Tournament do not have approximation ratios.

#### Reduce-Expand algorithm [6]

The Reduce-Expand algorithm (RE) is based on reducing sequences to *basic sequences*, which are sequences that have no adjacent characters of the same alphabet. For example, sequence "AACGG" can be reduced to basic sequence of "AG". The expand process tries to add characters into the common subsequence of the sequences, while preserving the common subsequence property. Using a process of reduce, auxiliary set and expand, this algorithm can produce short SCS on binary sequences as well as datasets with few sequences and more alphabets. The RE algorithm has an approximation ratio of *q *= |Σ| and has time complexity *O*(*q*^2+*α*^*kn*^2+*α*^log*n*) and space complexity *O*(*nk *+ *n*^2^), where *α *≥ 0 is a constant integer. It was shown in [6] that RE performs well on longer sequences (up to 300) with larger alphabets. However, the experimental studies were confined to datasets with relatively few sequences (*k *is small, up to about 20). For large SCS instances, the space and time requirements of RE may be a limiting factor.

**Figure 1:**
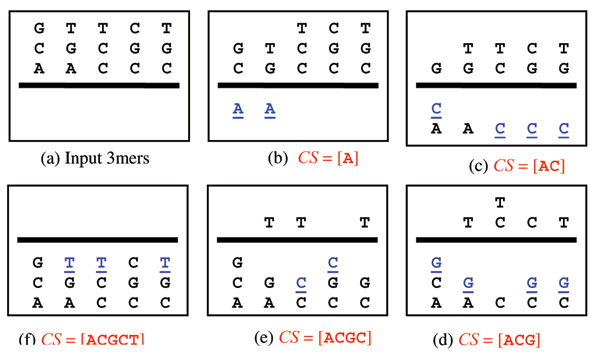
**A step-by-step illustration of the process of finding a common supersequence via deposition**. The characters above bar represents the sequences have yet to be deposited. The underlined blue characters are the characters that are deposited in the current step. The final result is CS = "ACGCT", which, for this example, is also the optimal result.

We next survey heuristic algorithms [10,11,13] that were designed specifically to target the SCS of DNA sequences – to be used for synthesis of DNA microarrays and oligo-arrays. To describe these methods, we adopt the notations used in [10], and used examples in DNA sequences. An example of sequence deposition is shown in Figure 1. After *t *cycles, a partial sequence has been synthesized. The height of each partially constructed sequence is defined as the number of bases in it. Indication of how much work that has been accomplished after *t *cycles is measured in terms of (i) the *Min-Height *(***MH***) – the height of the *shortest *partially constructed sequences after *t *cycles, or (ii) the *Sum-Height *(***SH***) – the *sum of the heights *of the partially constructed sequences.

#### Min-Height [10]

The Min-Height (MH) greedy method, denoted here by **MH**, selects a character that will *extend the shortest *partially constructed oligo by one base (thus, potentially increasing the minimum height). When there is a tie, we randomly pick one such character.

#### Sum-Height [10,11]

The Sum-Height (SH) greedy method, denoted by **SH**, selects a character that will result in the *largest increase *in the sum-height. This method is the same as the Majority Merge (MM) algorithm. Again, ties are broken randomly.

Both MH and SH are very fast algorithms, with time complexity of O(*qkn*) time. In general, they work well for DNA sequences.

#### Look-ahead extensions of SH and MH

A natural way to improve the fast greedy algorithms MH and SH is to apply a "*look-ahead*" strategy to it [11]. This strategy looks at a number of steps ahead before deciding which character(s) is the *best *to be added. More specifically, choose two integers *m *and *l *such that *l *≤ *m*. Then, the *look-ahead extension of the SH method *works as follows: (i) Examine all possible partial sequences that can be generated in *m *cycles; (ii) for each such generated partial sequences, compute the resulting sum height; and (iii) select the partial sequence that will result in the *largest increase of sum-height in m cycles*. Then, we extend the chosen sequence by *l *(≤*m*) characters. (It can be shown that extending by *l *characters (instead of *m*) gives the potential of obtaining even better increase in the sum-height after *m *cycles.) Here we break the tie arbitrarily. This look ahead algorithm is called the **(*m*, *l*)-look-ahead SH**, and is abbreviated to **(*m*, *l*)*-LA-SH***.

The look-ahead extension of MH is defined in a similar fashion, and is denoted by **(*m*, *l*)*-LA-MH***.

It is easy to see that an increase in *m *naturally leads to a shorter SCS, but at a cost of a substantial increase in computing time. Experimentation done in [11] indicated that LA-SH gives the best performance and that (3,1)*-LA-SH *gives the best trade-off between running time and quality of the SCS solution.

#### Look-Ahead Post Processing algorithm [11]

Finally, we recently proposed a Look-Ahead Post Processing (LAP) algorithm to solve the SCS problem on many DNA sequences that produce very good results for the case when the number of sequences *k *is large (*k *reaching 100,000) and sequence length *n *is relatively small (*n *up to 60). The LAP algorithm outperforms Alphabet, SH, and MH heuristics on these SCS instances.

#### Hubbell-Morris-Winkler algorithm [13]

The Hubbell-Morris-Winkler algorithm is another heuristic algorithm specifically designed for SCS of DNA oligos (short DNA sequences). The method consists of two key steps. First, it finds a shortest periodical strategy (one out of 4^4 ^periodical strategies) corresponding to the following string: (*XYZ*) (*XYZ*) ... (*XYZU*)(*XYZU*), where *X*, *Y*, *Z*, *U *denote the four different characters in some order. Second, examine each character in a cycle from the first to the last and remove the character if either (a) it is not needed by any oligo, or (b) it can be added in a later cycle. Hubbell-Morris-Winkler algorithm has comparable performance compared to our LAP algorithm on DNA sequences. *However, the algorithm is only suitable for sequences small alphabet size q such as DNA sequences*. For protein sequences (where *q *= 20), there are 20^20 ^periodical strategies and so the Hubbell-Morris-Winkler algorithm is not practical any more.

#### Other algorithms

Other SCS algorithms have also been proposed [14-16], but were not included in this study because they involve meta-heuristic search (genetic algorithms, ant colony optimization) and have very long running times. Thus, they are not practical for the large SCS instances considered in this paper.

### Performance ratio

To compare the performances of different algorithms across different instances of different sizes, we define the notion of *performance ratio*. For any instance *S*, the *performance ratio*, *R*_*A*_(*S*), *of algorithm A on instance S *is defined by *R*_*A*_(*S*) = |*CS*_*A*_(*S*)|/|*opt*(*S*)|, where *opt*(*S*) is an optimal SCS solution to the instance *S*.

However, |*opt*(*S*)| is unknown – it is not feasible to compute |*opt*(*S*)| since the SCS problem is NP-hard. Luckily, it is possible to compute a lower bound for |*opt*(*S*)|. We choose a small sample set, *SS*, of representative sequences in *S *and use an exact dynamic programming algorithm to compute *opt*(*SS*), the shortest common supersequence of the sample set *SS*. Clearly, |*opt*(*SS*)| is a lower bound on the optimum length, namely, |*opt*(*SS*)| ≤ |*opt*(*S*)|. Then the *performance ratio R*_*A*_(*S*) can also be upper bounded by the estimate *R'*_*A*_(*S*) = |*CS*_*A*_(*S*)|/|*opt*(*SS*)|. We shall use the estimated performance ratio bounds as a "metric" for comparing different algorithms later in this paper.

The gap between |*opt*(*S*)| and the computed lower bound, |*opt*(*SS*)| depends on how the sample set *SS *is chosen and also on its size. However, a large *SS *will render the computation infeasible. For DNA sequences, we use the sample set *SS *= {*SS*_A_, *SS*_C_, *SS*_G_, *SS*_T_} of 4 representative sequences from *S*, where each representative sequence *SS*_A _(and similarly, *SS*_C_, *SS*_G_, *SS*_T_) is a sequence in *S *that has the largest number of A's (and C, G, T, respectively). Then dynamic programming is applied on {*S*_1_, *S*_2_,...*S*_*q*_} to obtain a lower bound of the SCS length. For protein sequences with *q *= 20, we choose only top few (usually 4) such representative sequences in the sample set *SS*.

## Method

Since biological datasets usually contain many long sequences (namely *large SCS instances*), it is important to devise an effective algorithm that works well on large SCS instances (for both DNA and protein sequences). Among the existing heuristic algorithms, several of them (Greedy, Tournament, Reduce-Expand and to a less extend, Majority Merge) perform well on SCS instances where there are few long sequences (small *k *and large *n*). Several heuristic algorithms (Majority Merge, Min Height and their Look-Ahead variants, and LAP) perform well on SCS instances with many short sequences (large *k *and small *n*). However, relatively few research studies have been carried out to see how they perform on large SCS instances dealing with many long sequences (where both *k *and *n *are large). In fact, Barone *et al*. had in [6], raised the question on "how to design efficient (both in terms of time and space) heuristic algorithm on many long sequences".

In our recent work [11], we have compared the performances of several algorithms (MH, SH, (3,1)-LA-SH, and LAP) on SCS instances where *k *is large, but *n *is relatively small. The good performance of our LAP algorithm [11], indicates that the post processing approach *may also be effective on large SCS instances *(such as large sets of long DNA and protein sequences). This paper discusses how we modify the LAP algorithm to solve large SCS instances.

### Our DR algorithm for large SCS instances

In the *Deposition and Reduction *algorithm that we proposed, there are two processes: in the *deposition process*, we first generate a ***template pool ***– a small set of *SCS templates *(or *templates*, in short). Each template is a common supersequence of the SCS instance *S*. The *reduction process *shortens these templates by attempting to remove some characters while preserving the common supersequence property. This uses the post processing procedure introduced in [11].

### Deposition process (template generation)

For the deposition process, we use two algorithms to produce candidate templates that will be included in the **template pool**. Clearly, the performance of the algorithm is dependent on obtaining good SCS templates. Based on results from our previous study [11], we have selected (3,1)-LA-SH as one of the algorithms used to generate one of the templates. Another template used was that generated by Alphabet (largely so that the algorithm will have a worst-case performance guarantee).

### Reduction process

**Figure 2:**
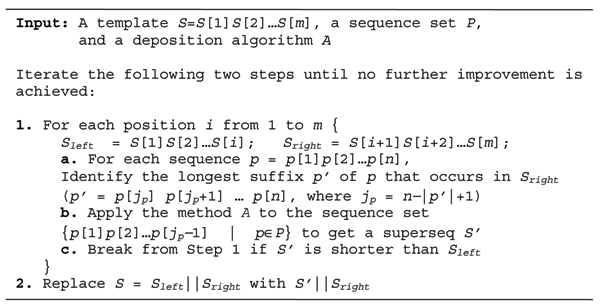
The procedure of the reduction process.

In the reduction process, we apply a reduction procedure to each SCS template in the template pool to obtain a shorter SCS. Finally, the shortest result obtained after this reduction process is selected as the final output of the algorithm. The reduction procedure we use is just a simple extension of the post-processing procedure in [11]. We give a brief description here. We are given a SCS template *S *= *S*[1]*S*[2]...*S*[*m*], with *m *characters (*S *is, of course, a common supersequence). The following approach, using *S *as a template and a method *A*, seeks to reduce the number of characters in *S*. The detailed algorithm is given in Figure 2.

In the current implementation of our Deposition and Reduction algorithm (DR), we have included the template generated by the Alphabet algorithm. Thus, our DR algorithm also has an approximation ratio of *q *= |Σ|. However, in practice, our experiment show the results from the (3,1)-LA-SH template is much better than those obtained from Alphabet and the performance ratios of the DR algorithm is much lower than *q*.

The time complexity of the deposition process is *O*(*q*^3^*kn*), the time complexity of the reduction process is *O*(*q*^3^*kn*^2^), so the total time complexity of the Deposition and Reduction algorithm is *O*(*q*^3^*kn*^2^). This is larger than that of Alphabet algorithm, but smaller than Greedy algorithm and Tournament algorithm where *q *is not very large and *k *is large. It is also smaller than that of the Reduce-Expand algorithm, especially when *n *is large. The space complexity of the Deposition and Reduction algorithm is *O*(*q*^3^*kn*).

## Results

Our post process algorithm is written in Java and Perl. The experiments are performed on a PC with 3.0 GHz CPU and 1.0 GB memory, running on a Linux operating system. We have selected Alphabet (ALPHA) [6], Reduce-Expand (RE) [6], Tournament (TOUR), Greedy (GRDY) [12] and Majority Merge (MM) [8] algorithms, as well as Lower Bound (LB) for comparison with our Deposition and Reduction (DR) algorithm on large SCS instances. We also used abbreviations defined above for the different heuristic algorithms. We note here that the RE algorithm was not included in the comparative study of large SCS instances as the RE algorithm took too long to run on these large SCS instances. Instead, we compare the RE algorithm with our DR algorithm directly on smaller SCS instances in the subsection on "Comparison with Reduce-Expand Algorithm".

For the large SCS instances, we use both simulated sequences as well as real (DNA and protein) sequences. It is easy to see that results on datasets with sequences of different lengths are similar to those results on datasets with sequences of same lengths. Therefore, in this study, we have only used simulated sequences of same length in the dataset, and also truncated real sequence to same lengths in each datasets. In the tables of experimental results below, *k *denotes the number of sequences, and *n *denotes the length of the sequence. For DNA sequences, the size of alphabet *q *= 4, while for protein sequences *q *= 20.

### Results on simulated sequences

We first carry out comparative study on simulated DNA sequences. For each value of *k *= 100, 500, 1000, 5000, and *n *= 100, 1000, we generated 10 random datasets of DNA sequences. These datasets are shown in Tables 1 and 2 where each row represents the composite result for the 10 instances. For each row, we also compute the average lower bound (denoted by LB in Tables 1 and 2) over the 10 instances. Then, each algorithm, *A*, is run on these 10 instances to get an average (and standard deviation) of |*CS*_*A*_(*S*)|, the length of the SCS. The estimated performance ratios of the different algorithms (denoted by Ratio in Tables 1 and 2) are obtained by dividing the average SCS lengths by the lower bound LB. As mentioned earlier, these are *upper bounds *on the true performance ratios of the algorithms.

In Table 1, we first study the effect of Deposition process and Reduction process in our DR algorithm. To this end, we define *CS*_*D *_and *CS*_*R *_to be the common supersequences obtained after the Deposition and Reduction process, respectively. From Table 1, we observed that both the Deposition and the Reduction processes are effective. The Deposition process always output templates with *|template*_*D*_*| */*|opt(S)| *less than 1.8 for both long (*n *= 1000) and short (*n *= 100) sequences. In the following tables, we will see that this is much better than results of Alphabet, indicating the superiority of (3,1)-LA-SH template. The Reduction process can further reduce the lengths of the results by between 2 to 6 characters for *n *= 100, and by about 20 characters for *n *= 1000, and the performance ratios are correspondingly reduced. The standard deviations for the results of both Deposition and Reduction processes are not big, indication that both the Deposition process and the Reduction process give stable results.

In Table 2, we then compared the various heuristic algorithms – Alphabet, Tournament, Greedy, Majority Merge (or Sum Height) with our DR algorithm on these *simulated *DNA datasets. To assist the comparison, we have also included the lower bound (LB), and the average estimated performance ratios.

The results in Table 2 show *very clearly *that for the datasets with large *k *and *n*, the DR algorithm consistently gives the best results, followed quite closely by MM, while algorithms TOUR and GRDY are quite a bit worse. Generally, we observe that the length of the SCS obtained increases with the number of sequences, which is to be expected.

The performances of the algorithms on medium length sequences (*n *= 100) also differ slightly from those for long sequences (*n *= 1000). For *n *= 100, our DR algorithm produces results that are, on average, shorter than those of MM by 13.5 characters, by 49.4 characters than those of GRDY, and by 54 characters than those of TOUR. For long sequences (*n *= 1000), the difference are more pronounced, by 55.6 characters for MM, by 539 characters for GRDY, and by 557 characters for TOUR.

Similar to the results observed in [1], the performance ratios obtained by all the algorithms are quite a bit below the worst-case ratio of 4 for the trivial Alphabet algorithm. The standard deviation results in Table 2 also show that DR algorithm is relatively stable – more stable than GRDY and TOUR, but not as stable the MM algorithm.

We have not done a similar comparison on simulated protein sequences, but we expect that the relative performances of these algorithms on random protein sequences would be similar to those for random DNA sequences. In the next section, we show results on real DNA and protein sequences.

### Results on real biological sequences

In this section, we compared the algorithms Alphabet and MM with our DR on datasets obtained from real DNA and protein sequences. For this study, we have to exclude the GRDY and TOUR algorithms since their performances are much worse than MM or DR, and they are also very time-consuming on these datasets.

For this experimental comparison, we have randomly selected DNA sequences from the NCBI viral genomes [17]. These DNA sequences are truncated so that they have lengths (*n*) of 500 and 1000 and combined to obtained many datasets that are grouped into four cases (DNA-1 to DNA-4) with *k *= 100 and 500, and 10 randomly selected datasets for each setting, as shown in Table 3. The protein sequences (with *q *= |Σ| = 20) are from SwissProt [18] and these have been truncated to length 500 and we have used *k *= 100, 500, and 1000 to truncate datasets, and 10 randomly selected datasets for each setting. These datasets are grouped into three cases (PROT-1 to PROT-3) as shown in Table 3. Both real DNA and protein sequences datasets are available as additional supplemental materials.

The comparison results for MM and DR algorithms on these selected DNA and protein sequences are shown in Table 3. The results for real DNA sequences are similar to those for simulated DNA sequences. Again, the results clearly show that DR consistently outperform the MM algorithm for both type of sequences. The performance ratio |*CS*_*DR*_(*S*)|/|*opt*(*S*)| of DR algorithm is about 2.1 for DNA sequences with length 500 and 1000, and 6.0~7.51 for protein sequences with length 500. These are much less than the approximation ratios. We also observe that the length of the SCS obtained increases with the number of sequences.

Comparing Table 3 with Table 2, we also observed that the performance ratios for MM and DR are generally a little bigger for real DNA sequences as compared to simulated DNA sequences for similar values of *k *and *n*. This may be attributed to the fact that the real DNA sequences are selected from different viral genomes and thus, there is high variance in the GC contents and this may have resulted in longer SCS. Whereas, in the simulated DNA sequences, the GC content is predefined for each of the simulated DNA sequences datasets and this may have resulted in the shorter SCS obtained by the algorithms. This observation is also consistent with results in [11].

The results for protein sequences show a similar trend as those for DNA sequences. Note that the approximation ratio for the Alphabet algorithm is 20 for protein sequences. Our results show that for DR on protein sequences with *n *= 500, the performance ratio is smaller than those for MM. The difference in the performance ratios of DR and MM is larger for protein sequences that have a larger alphabet. In terms of the length of the SCS result, DR obtains SCS results that are between 300 to 450 characters shorter compared to those obtained by MM.

The standard deviations observed in Table 3 for DNA and protein sequences are relatively small, again indicating that both MM and DR algorithms give relatively stable performance.

### Comparison with Reduce-Expand algorithm

The Reduce-Expand (RE) algorithm [1] is currently one of the best algorithms for the general SCS problem for small SCS instances. Because of the high running time of the RE algorithm, we have only compared RE with our DR algorithm on simulated datasets with relatively few, short DNA sequences (*q *= 4, *k *= 5, 10, 50, 100, *n *= 10, 100, and with 10 randomly generated datasets for each setting), as well as small datasets of real DNA and protein sequences. The results on simulated datasets are shown in Table 4.

The results in Table 4 indicate that DR is only comparable to (and for *n *= 100, even longer than) RE in the lengths of the results when there are only 5 sequences, but outperform RE in the lengths of the results when the number of sequences is over 10. The more sequences, and the longer the sequences, the larger differences between the length of the results of DR and RE. Especially for longer (length of 100) sequences, the results of DR can be about 5 to 20 characters shorter than the results of RE.

We have also compared the algorithms Alphabet and RE with our DR on datasets obtained from real DNA and protein sequences. The sequences in datasets (DNA-5, DNA-6, PROT-4 and PROT-5, and 10 randomly selected datasets for each setting) are shorter from those in Table 3, and the results are shown in Table 5.

The results on real DNA and protein sequences are consistent with the results on simulated sequences. The DR algorithm can outperform the RE algorithm by about 10 characters for DNA sequences of length 100. The SCS results of the DR algorithm are also shorter than the results of the RE algorithm by more than 20 characters on protein sequences. The more sequences, and the longer the sequences, the larger differences between the length of the results of the DR and RE algorithm.

### Computational efficiency

Our Deposition and Reduction (DR) algorithm has time complexity of O(*q*^3^*kn*^2^) and space complexity of O(*q*^3^*kn*). In our experiments, the DR algorithm is very fast, even for large SCS datasets – for example, for 1000 sequences of length 1000 each, it take an average of 5–10 minutes per instance. In comparison to existing algorithms, it is slower than Majority Merge, Min Height (less than 1 minute), but it is much faster than Greedy and Tournament algorithms (both of which need more than 60 minutes). The DR algorithm is also much faster than the Reduce-Expand algorithm (our Perl implementation). For a simulated DNA dataset with 100 sequences each of length 100, the DR algorithm can get result in less than 10 seconds, while the Reduce-Expand algorithm needs more than 10 minutes. The actual computer memory used by the DR algorithm is about 50 M for some of our very large datasets; Majority Merge needs about 10 M for these same datasets. The software is available upon request, and the web services portal will be available soon.

## Conclusion

In this paper, we have proposed a Deposition and Reduction (DR) algorithm for solving large SCS instances. The DR algorithm is composed of the Deposition process to generate good templates, and the Reduction process to reduce templates from template pool to get short result. These processes are shown to be powerful for the SCS problem.

We have compared the performance of our DR algorithm with some of the best heuristic algorithms on different sequences datasets, especially on many long sequences. The DR algorithm has superior performance than Alphabet, Tournament, Greedy and Majority Merge algorithms in practice, especially on many long sequences. It also outperforms the Reduce-Expand algorithm for sequences of length 50~100. The DR algorithm is also very efficient in time and space, which partially answer the question that Barone et al. raised [6] about how to design efficient (both in terms of time and space) heuristic algorithm on many long sequences.

The Deposition and Reduction algorithm is an extension of our previous study for SCS problem on DNA oligos [11]. To our best knowledge, our Deposition and Reduction is one of the best heuristic algorithms (both in terms of performance ratios and in terms of time and space needed) for the SCS problem on biological sequences such as the DNA and protein sequences, especially for datasets with many long sequences. We believe that the use of the Deposition and Reduction algorithm can facilitate the biological sequencing process for bioinformatics researches.

There are many other post process strategies for the SCS problem, such as better look ahead strategies, which may lead to better performance. We are currently trying to further analyze these strategies for SCS problems by comparing the results of different strategies.

As a general computational framework, this Deposition and Reduction algorithm can also be applied on more general applications such as text comparison and compression, query optimization and scheduling. We will also work on these more general problems in the future.

## Supplementary Material

Additional file 1 **Real DNA sequences **DNA sequences are obtained from NCBI Viral Genomes, Accessed on 01/01/2006. . Sequences are randomly selected from different genome sequences. The specific length and number of sequences are specified for each dataset. There are 60 randomly selected datasets. File name indicate the number of sequences, sequence length, and dataset number. For example, file name "DNA_N100_K500_5" indicate that it is DNA sequences dataset, with 500 sequences, each truncated to length of 100. And this is number 5 datasets for this setting. Each sequence is represented as a row in the dataset file. Click here for file

Additional file 2 **Real Protein sequences **PROTEIN sequences are obtained from Swiss-Prot Release 45.5 of 04-Jan-2005. . Sequences are randomly selected from different protein sequences. The specific length and number of sequences are specified for each PROTEIN dataset. There are 60 randomly selected datasets. File name indicate the number of sequences, sequence length, and dataset number. For example, file name "PROTEIN_N100_K500_5" indicate that it is PROTEIN sequences dataset, with 500 sequences, each truncated to length of 100. And this is number 5 datasets for this setting. Each sequence is represented as a row in the data file. Click here for file

## Figures and Tables

**Table 1:** The results of the Deposition process and the Reduction process.

***Sequences***		***Length of CS (averaged over 10 instances)***				
***k***	***n***	***LB***	**|*CS*_*D*_|**	***R'*_*D*_**	**|*CS*_*R*_|**	***R'***_***R***_

100	100	158.0	267.8 (7.1)	**1.69**	**264.5 (6.8)**	**1.67**
500	100	160.6	274.9 (7.3)	**1.71**	**273.0 (7.1)**	**1.70**
1000	100	161.3	277.1 (6.0)	**1.72**	**275.6 (6.2)**	**1.71**
5000	100	162.9	281.6 (4.0)	**1.73**	**279.9 (4.2)**	**1.72**
100	1000	1441.8	2495.4 (50.6)	**1.73**	**2480.2 (55.5)**	**1.72**
500	1000	1457.6	2543.9 (48.6)	**1.75**	**2527.2 (52.1)**	**1.73**
1000	1000	1472.3	2557.5 (45.1)	**1.74**	**2540.0 (47.4)**	**1.73**
5000	1000	1481.6	2566.6 (35.1)	**1.73**	**2548.3 (39.7)**	**1.72**

Each row of the table represents 10 randomly generated instances. For each row, we list the average value for LB (the lower bound), |*CS*_*D*_|, and |*CS*_*R*_| (the length of the common supersequences after Depostion and Reduction), their standard deviations (in parenthesis), and the estimated performance ratios *R'*_*D *_and *R'*_*R *_(|*CS*_*D*_|/LB and |*CS*_*R*_|/LB)

**Table 2:** A comparison of the lengths of the SCS results obtained by different algorithms on simulated DNA sequences.

***Sequences***		***Length of CS (averaged over 10 instances)***										
***k***	***n***	***LB***	***ALPHA***	***Ratio***	***TOUR***	***Ratio***	***GRDY***	***Ratio***	***MM***	***Ratio***	***DR***	***Ratio***

100	100	158.0	400	2.53	304.6 (15.3)	1.93	303.9 (11.9)	1.92	276.8 (5.5)	1.75	**264.5 (6.8)**	**1.67**
500	100	160.6	400	2.49	329.0 (18.2)	2.05	321.6 (9.1)	2.00	286.4 (7.7)	1.78	**273.0 (7.1)**	**1.70**
1000	100	161.3	400	2.48	335.4 (19.9)	2.08	329.0 (17.8)	2.04	289.1 (8.2)	1.79	**275.6 (6.2)**	**1.71**
5000	100	162.9	400	2.46	339.8 (21.2)	2.09	336.2 (21.2)	2.06	294.8 (10.6)	1.81	**279.9 (4.2)**	**1.72**
100	1000	1441.8	4000	2.77	2936.7 (146.6)	2.04	2921.5 (143.4)	2.03	2547.1 (24.7)	1.77	**2480.2 (55.5)**	**1.72**
500	1000	1457.6	4000	2.74	3049.6 (150.0)	2.09	3043.6 (145.2)	2.09	2578.0 (23.3)	1.77	**2527.2 (52.1)**	**1.73**
1000	1000	1472.3	4000	2.72	3142.3 (176.9)	2.13	3115.5 (173.6)	2.12	2590.9 (26.5)	1.76	**2540.0 (47.4)**	**1.73**
5000	1000	1481.6	4000	2.70	3194.5 (221.9)	2.16	3172.8 (199.0)	2.14	2602.1 (25.6)	1.76	**2548.3 (39.7)**	**1.72**

The average and standard deviation (in parenthesis) over 10 randomly generated instances are given. The estimated performance ratios are also given for each algorithm.

**Table 3:** The comparison of the lengths of the SCS results obtained by different algorithms on selected DNA and protein sequences.

***Sequences***			***Length of CS (averaged over 10 instances)***						
	***k***	***n***	***LB***	***ALPHA***	***Ratio***	***MM***	***Ratio***	***DR***	***Ratio***

***DNA sequences***									

DNA-1	100	500	686.6	2,000	2.91	1359.7 (18.7)	1.98	**1346.4 (19.6)**	**1.96**
DNA-2	500	500	689.5	2,000	2.90	1430.5 (22.1)	2.07	**1420.7 (18.1)**	**2.06**
DNA-3	100	1000	1361.0	4,000	2.94	2698.0 (39.9)	1.98	**2675.7 (37.2)**	**1.97**
DNA-4	500	1000	1364.4	4,000	2.93	2822.3 (43.4)	2.07	**2769.1 (32.7)**	**2.03**

***Protein sequences***									

PROT-1	100	500	800.6	10,000	12.49	5312.7 (81.9)	6.64	**4846.3 (73.9)**	**6.05**
PROT-2	500	500	803.5	10,000	12.45	5935.6 (61.8)	7.39	**5548.6 (54.3)**	**6.91**
PROT-3	1000	500	809.8	10,000	12.35	6082.4 (44.9)	7.51	**5734.0 (43.9)**	**7.08**

The average and standard deviation (in parenthesis) are given. The estimated performance ratios are also given for each algorithm.

**Table 4:** The comparison of the lengths of the SCS results between RE and DR on simulated DNA sequences.

***Sequences***		***Length of SCS (averaged)***		
***k***	***n***	***ALPHA***	***RE***	***DR***

5	10	40	20.45 (0.53)	**20.05 (1.36)**
10	10	40	26.04 (0.93)	**25.00 (1.76)**
50	10	40	35.00 (1.00)	**29.90 (1.55)**
100	10	40	34.69 (0.97)	**30.10 (1.30)**
5	100	400	188.08 (5.55)	**195.60 (6.27)**
10	100	400	229.28 (7.61)	**224.20 (6.28)**
50	100	400	286.12 (13.34)	**257.85 (6.80)**
100	100	400	281.95 (10.86)	**264.45 (6.86)**

In each cell, the average and standard deviation (in parenthesis) over 10 randomly generated instances are given.

**Table 5:** The comparison the lengths of the SCS results between RE and DR on selected DNA and protein sequences.

	***k***	***n***	***ALPHA***	***RE***	***DR***
***DNA***					

DNA-5	100	100	400	301.04 (8.85)	**284.40 (4.54)**
DNA-6	500	100	400	308.87 (7.61)	**296.80 (3.66)**

***Protein***					

PROT-4	100	100	2,000	1028.90 (35.54)	**1008.70 (14.29)**
PROT-5	500	100	2,000	1232.10 (31.03)	**1199.80 (13.80)**

In each cell, the average and standard deviation (in parenthesis) over 10 randomly generated instances are given.
